# A case report of a giant right ventricular outflow tract in a young man

**DOI:** 10.1097/MD.0000000000005313

**Published:** 2016-11-11

**Authors:** Die Hu, Dao-Quan Peng, Xiang-Ping Li, Bi-Lian Yu

**Affiliations:** Department of Cardiovascular Medicine, The Second Xiangya Hospital, Central South University, Changsha, Hunan, People's Republic of China.

**Keywords:** cardiomyopathy, localized constrictive pericarditis, right heart failure

## Abstract

**Introduction:**

Localized pericardium restriction is a rare disease and likely to be unrecognized owing to the atypical manifestation, even after diagnostic avenues are exhausted. Recognizing the red flags of the disease could timely spark a preliminary suspicion of the disease and thus contribute to the early application of relevant examinations.

**Case presentation:**

We will here report a case of a 21-year-old young man with a giant right ventricular outflow tract. He was presented to our hospital for further evaluation of progressive right heart failure which had been previously diagnosed as cardiomyopathy. Unlike patients with right heart failure owing to the restrictive cardiomyopathy, our patient's tissue Doppler revealed an increased early diastolic septal mitral annular velocity. In addition, the disproportion between the severity of right heart failure and the degree of myocardial dysfunction could not be completely explained by other myocardial disease, suggesting that alternative diagnosis of the patient should be sought. Subsequently, cardiac computed tomography, which revealed the focally calcific pericardium encircling the left ventricle, gave us a clue to the diagnosis of localized constrictive pericarditis. Cardiac catheterization, showing the “dip and plateau” sign, further confirmed this diagnosis. The patient underwent successful pericardiectomy. Nowadays, he is able to undertake ordinary physical activity.

**Conclusion:**

Localized constrictive pericarditis should be suspected in patients for whom the severity of heart failure and deformity of heart might not be completely explained by valvular heart disease or myocardial disease.

## 1 Introduction

Constrictive pericarditis, as a curable disease, is comparatively common in clinical practice. It is characterized by impaired diastolic filling of the ventricles owing to reduced compliance of a stiff pericardium. Though new imaging investigation could facilitate accurate identification of constrictive pericarditis, this disorder presenting with atypical manifestations still remains unsuspected owing to overlooking integrated use of related examinations, especially for deformative shape of heart without obvious structural and hemodynamic features of pericardial constriction in echocardiography.^[1]^

According to related literatures, the cases of localized constrictive pericarditis are rare and easy to be misdiagnosed as other diseases, such as aneurysm, apical pseudo-ballooning, and so on.^[2–4]^ Now, we report a case of localized constrictive pericarditis presenting with giant right ventricular outflow tract (RVOT), masquerading as cardiomyopathy.

## 2 Case report

**Figure 1:**
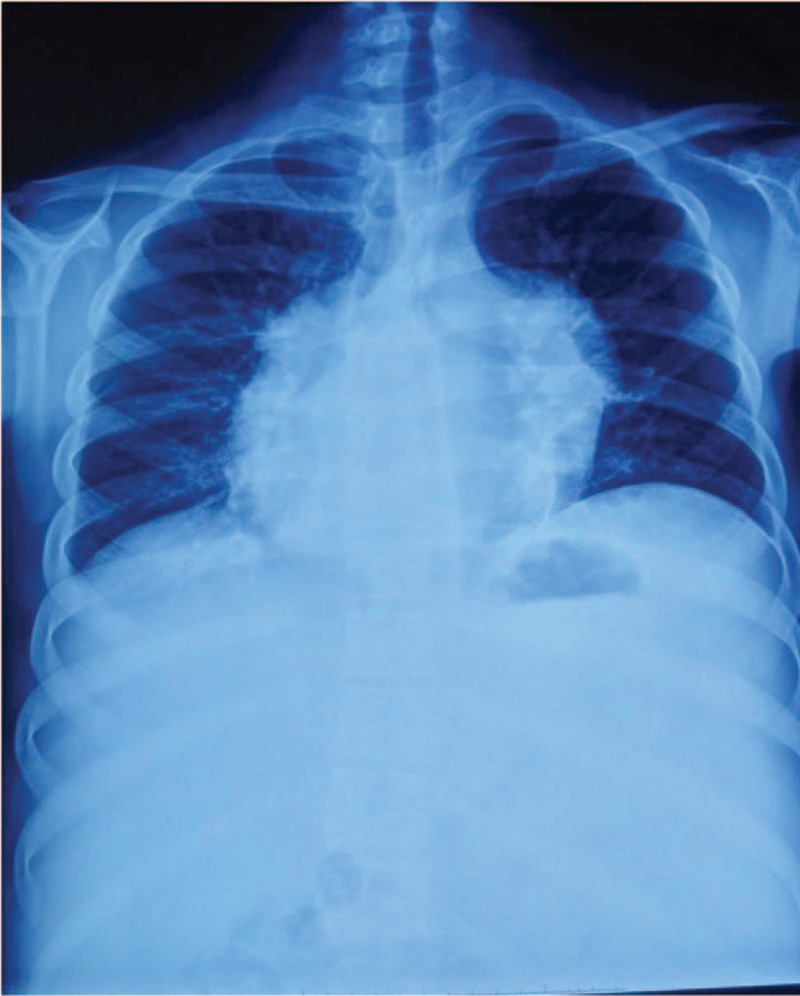
Chest x-ray showed the enlarged and irregular ball-like cardiac silhouette.

A 21-year-old male patient was admitted to our institution for recurrent symptoms of right heart failure for more than 10 years, associated with 2 episodes of syncope. On the basis of physical examination, he was found to have marked jugular venous distension, abdominal fullness with significant ascites, and slightly lower extremity edema. No pericardial knock or third heart sound was detected on auscultation, and the lungs were clear. His medical history was unremarkable with the exception of chronic hepatitis B that had been well controlled. The diuretic was taken for relieving the recurrent edema and dyspnea. There was no family history of premature cardiac symptoms or sudden death. Results of laboratory tests, including blood biochemistry, renal, and liver function, were within normal limits, except for the mild anemia with hemoglobin of 95 g/L. The serum N-terminal probrain natriuretic peptide of 2467 pg/mL was elevated (normal range of <900 pg/mL). Electrocardiogram demonstrated sustained atrial fibrillation with the inversion of T wave at precordial derivations of V1 to V3, and no epsilon waves. A chest X-ray showed an enlarged and irregular ball-like cardiac silhouette (Fig. 1).

**Figure 2:**
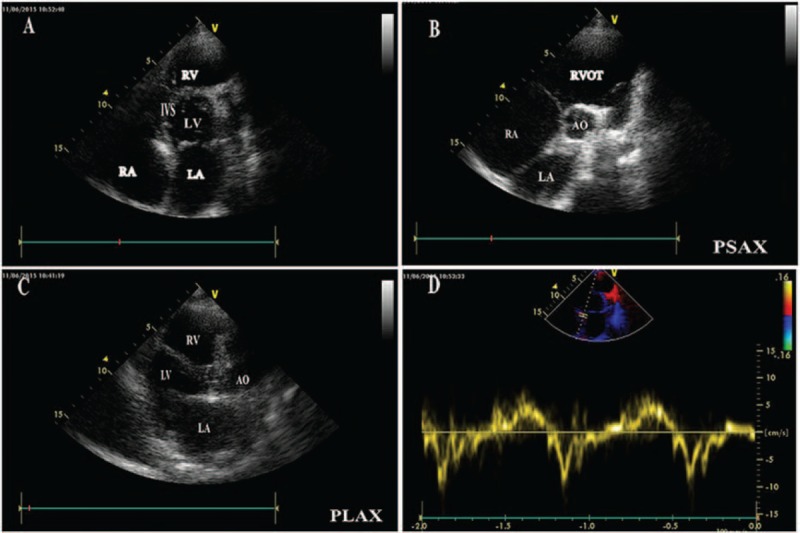
Transthoracic 2-dimensional echocardiogram images. (A) Four-chamber view shows the right ventricular dilation (right ventricle = 36 mm), biatrial enlargement, especially the massive right atria (left atrium = 48 mm, right atrium = 59 mm). In contrast, the left ventricle (LV) is smaller than right atria (LV = 44 mm). The whole view shows a special cardiac contour. (B and C) Both parasternal short-axis and long-axis views show right ventricular dilation associated with the markedly enlarged right ventricular outflow tract = 47 mm. (D) The tissue Doppler showed that the early diastolic septal mitral annular velocity was 10 cm/s.

**Figure 3:**
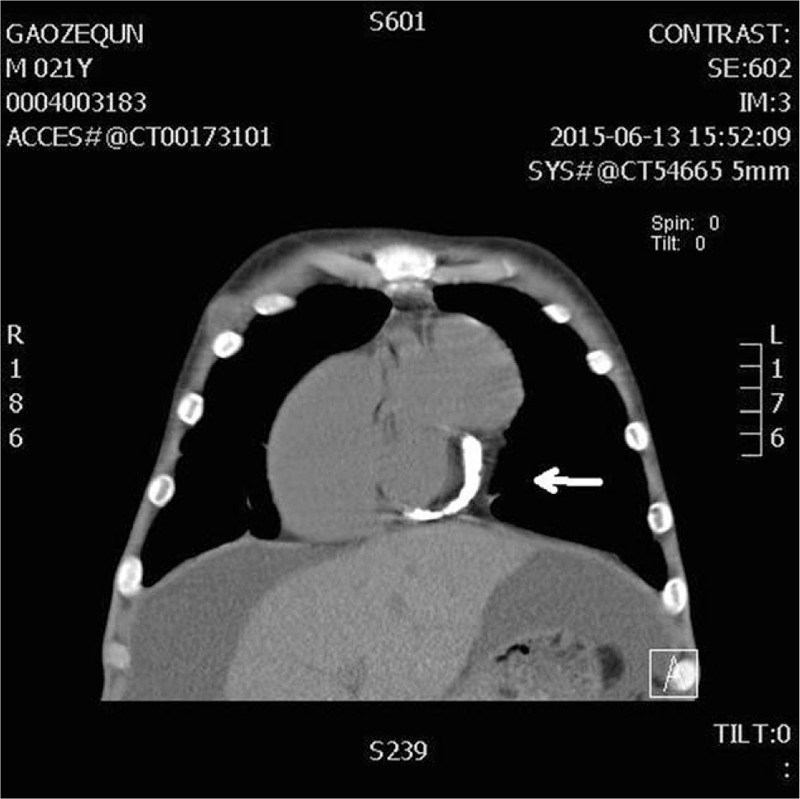
Computer tomography scans showing focally pericardial calcification in a ring-like pattern encircling the left ventricle (white arrows).

Echocardiography revealed the right ventricular dilation associated with a giant RVOT = 47 mm, the biatrial enlargement, especially the massive right atrium = 59 mm, and absence of the ventricular hypertrophy (Fig. 2A–C). There were slight regurgitations at mitral and tricuspid valves. Ejection fraction (EF) was noted to be impaired (EF = 40%). However, the tissue Doppler revealed an increased early diastolic septal mitral annular velocity (Fig. 2D). Subsequently, cardiac computed tomography (CT) not only confirmed the above echo findings of remarkably dilated RVOT, but also incidentally showed the focally calcific pericardium in a ring-like pattern encircling the left ventricle (LV), which provided us with the clue to the suspicion of localized constrictive pericarditis (Fig. 3). Cardiac catheterization revealed the discordance of the ventricular pressure tracings with respiration and the “dip and plateau” sign of ventricular diastolic pressure, thus confirming the diagnosis of constrictive pericarditis.

**Figure 4:**
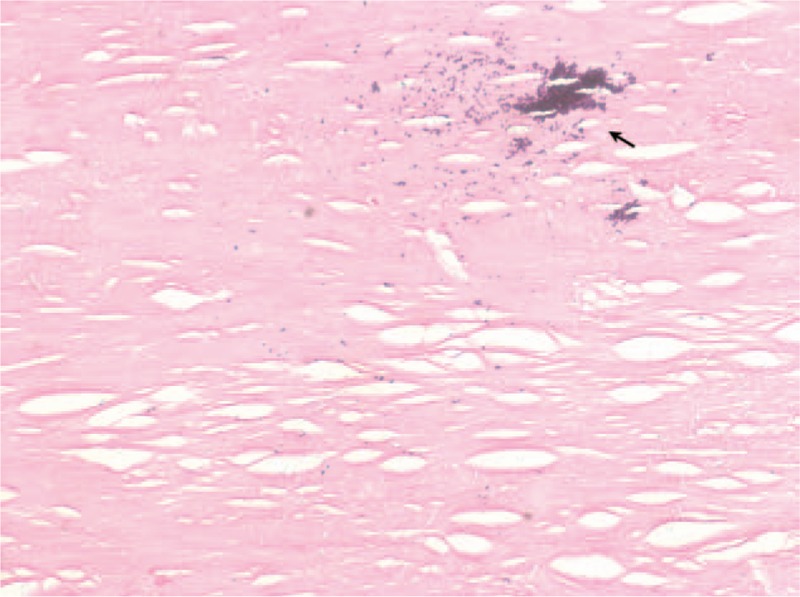
Histopathologic study of the resected pericardium exhibits significant fibroplasia and hyalinization associated with small foci of calcification (black arrows). (Hematoxylin eosin stain, magnification ×200).

On the basis of the diagnosis, the patient underwent pericardiectomy. In the operation, dense, fibrothickened, and calcified pericardium was found to mainly encircle the LV, partial right ventricle (RV), and atrioventricular groove, which could cause regional compression and constrictive effects. Hence, the right ventricular outlet tract, overlying normal pericardium, was dilated gradually for higher intraventricular filling pressure. The pathology of resected pericardium showed significant fibroplasias and hyalinization with mild calcification (Fig. 4). Ultimately, the patient was uneventfully discharged after surgery. Nowadays, he is able to undertake ordinary physical activities, and his echocardiography follow-up after the pericardiectomy suggests improved cardiac function.

## 3 Discussion

To the best of our knowledge, our case is the first to demonstrate localized constrictive pericarditis presenting with giant RVOT. Initially, the constrictive pericarditis was concealed due to atypical echo findings of constrictive pericarditis. Because of the 2 syncopal episodes together with the lesion of RV, diagnosis of the arrhythmogenic right ventricular cardiomyopathy (ARVC) was considered. However, this patient's cardiac CT, which revealed obvious pericardial calcification, provided us with a clue to the suspicion of localized constrictive pericarditis instead of ARVC. Correlated with the patient's symptoms and invasive hemodynamics of physiological constriction, the diagnosis of localized constrictive pericarditis was confirmed.

Constrictive pericarditis is characterized by impaired diastolic filling of ventricles due to the chronic fibrous thickening, or calcification of the pericardium, or both. So, when a patient has unexplained systemic venous congestion, constrictive pericarditis should be considered. In patients with constrictive pericarditis, the echocardiography generally demonstrates pericardial hyperechogenicity, abnormal interventricular septum motion, respiratory variation in transmitral flow, the dilated inferior vena cava without inspiratory collapse, and preserved or increased mitral annular e′ evaluated by the tissue Doppler.^[5]^ These features may help to discriminate it from other causes of right heart failure, such as tricuspid valve disease, restrictive cardiomyopathy, or pulmonary hypertension. Besides, cardiac CT and magnetic resonance imaging, as valuable complementary imaging methods, can allow more excellent visualization of the pericardium and thus facilitate detecting an abnormal pericardium missed on a transthoracic echocardiogram. However, some focal thickness or calcification may not always imply constriction, or conversely, constrictive pericarditis can also present itself without any obvious pericardial calcification or thickening. No single method is completely reliable. Invasive hemodynamics, which can reflect pathophysiological abnormality, plays a pivotal role in confirming the diagnosis of constriction in patients whose noninvasive evaluation is equivocal.

Admittedly, multiple methods can be used to identify the constrictive pericarditis. This disease with atypical manifestations still remains unsuspected owing to overlooking integrated use of related examinations. Compared to diffuse constrictive pericarditis, localized constrictive pericarditis tends to have a special cardiac deformation because of the long-standing and higher intraventricular filling pressure in the regions without pericardial constriction. According to the bibliography, owing to anatomical distribution of the constrictive effects, localized constrictive pericarditis is readily regarded as an aneurysm or cardiac apex diverticulum at first.^[2–4]^ In this case, the obvious dilated right ventricular outlet tract is partially related to the childhood onset of the localized pericardium restriction, which can result in the deformity of a developing heart. The long-term restriction of the heart can also impair the systolic function as seen in this patient.

## 4 Conclusion

In summary, the early diagnosis of constrictive pericarditis is of paramount clinical significance. However, localized pericardium restriction is probably unrecognized owing to atypical symptoms, even after the diagnostic avenues are exhausted. Therefore, recognizing the red flags of the disease facilitates the early diagnosis. As our case shows, focal constrictive pericarditis should be suspected in patients for whom the severity of heart failure and deformity of heart might not be completely explained by valvular heart disease or myocardial disease.
